# A Mixed-Methods Investigation of First-Year Medical Students’ Professionalism Competency Development over the Gross Anatomy Course

**DOI:** 10.1007/s40670-024-02204-8

**Published:** 2024-11-14

**Authors:** Emily M. Porta-Miller, Jennifer Brueckner-Collins

**Affiliations:** Department of Anatomical Sciences and Neurobiology, University of Louisville School of Medicine, Louisville, KY USA

**Keywords:** Competency-based medical education, Preclinical medical education, Competency assessment, Professionalism, First-year medical students, Gross anatomy education

## Abstract

**Background:**

While competency-based medical education (CBME) and competency assessment in clinical training are well documented, they are less commonly included in preclinical medical education. The gross anatomy laboratory is an opportune setting to incorporate competency assessment and reflection during preclinical medical education. This mixed-methods study determined how first-year medical student assessments of professionalism skills in the gross anatomy lab change over time and analyzed student reflections to contextualize the experiences they had developing the Professionalism competency.

**Methods:**

First-year medical students at the University of Louisville completed self- and peer-assessments using the Professionalism Assessment Scale (PAS) at the beginning and end of their gross anatomy course (*n* = 83). The students also completed three competency development portfolio (CDP) entries throughout the course (*n* = 83). Qualitative thematic analysis with grounded theory was used to analyze comments related to professionalism skill development in the CDP reflections during the course.

**Results:**

There was no statistical difference in PAS self-assessment scores from Time 1 (*M* = 4.81, *SD* = .209) to Time 2 (*M* = 4.85, *SD* = .217), *p* = .108. There was a statistically significant increase in PAS-peer-assessment scores from Time 1 (*M* = 4.89, *SD* = .165) to Time 2 (*M* = 4.93, *SD* = .127), *p* = .005. Thematic analysis of CDPs revealed that students believed they developed interpersonal relations/social skills, responsibility skills, and gross anatomy lab-specific skills over the gross anatomy course.

**Conclusions:**

The Professionalism competency is inherently present and able to be assessed in the gross anatomy lab context for first-year medical students.

## Introduction

“Professionalism” represents one of the six general competencies endorsed by the Accreditation Council of Graduate Medical Education (ACGME) and American Board of Medical Specialties (ABMS) that is used to evaluate medical residents and reflects a skill necessary of a practicing physician [1]. The Association of American Medical Colleges (AAMC), American Association of Colleges of Osteopathic Medicine (AACOM), and ACGME are currently sponsoring an initiative to develop a common set of foundational competencies for use in undergraduate medical education (UME) programs within the USA as a comprehensive effort to improve medical students’ transition to residency [2]. Professionalism will be reflected within these foundational competencies, which will intend to represent minimum competencies for all medical students, regardless of degree type or eventual specialty of practice [2]. The University of Louisville School of Medicine (ULSOM) is already including Professionalism Skills and Personal and Professional Development as two of the core competencies within the school of medicine program objectives which all graduates should be able to demonstrate by graduation. Within these competencies, graduates should be able to exhibit behaviors of professionalism required for working in a stressful and team-oriented environment; form a healthy professional identity that adheres to the standards of the medical profession, including respect for all persons, compassion and empathy, trustworthiness, and integrity; set and revise personal and professional development goals based on participation in formal self-assessment and periodic reflection activities; and establish effective work habits, including organized and timely completion of required duties and assignments, etc. [3].

The AAMC Professional Task Force defined medical professionalism as “the enactment of the values and ideals of individuals who are called, as physicians, to serve individuals and populations whose care is entrusted to them, prioritizing the interest of those they serve above their own” [4]. The preclinical years of medical education can provide the foundational professionalism and ethics necessary to continue medical training, and the gross anatomy lab with cadaveric dissection offers a unique opportunity for first-year medical students to confront their “first patient” [4, 5]. Dissection-based gross anatomy labs provide implicit skills to develop basic elements of professionalism that are assessed during clinical years of medical education, but these skills must be recognized and taught in order to be evaluated [4]. It has been determined that no individual tool is able to reliably and effectively measure students’ professionalism; rather, a multi-tool approach should be used due to the situational and complex nature of professionalism [5, 6].

We agree with Escobar Poni and Poni [4] and Palmer et al. [5] and suggest the gross anatomy laboratory with dissection-based learning as an opportune venue to implement Professionalism competency assessment during the first year of undergraduate medical education (UME) using self- and peer-assessments and reflective portfolio pieces. This mixed-methods investigation of competency development is intended to (1) quantitatively determine whether first-year medical students’ self-assessments of the Professionalism competency in the gross anatomy lab context exhibit change over time; (2) quantitatively determine whether first-year medical students’ peer-assessments of the Professionalism competency in the gross anatomy lab context exhibit change over time; and (3) qualitatively analyze and contextualize student reflections on their baseline status, progress, and development of their Professionalism competency throughout the gross anatomy course.

## Materials and Methods

### Overview of Methodology

**Fig. 1:**
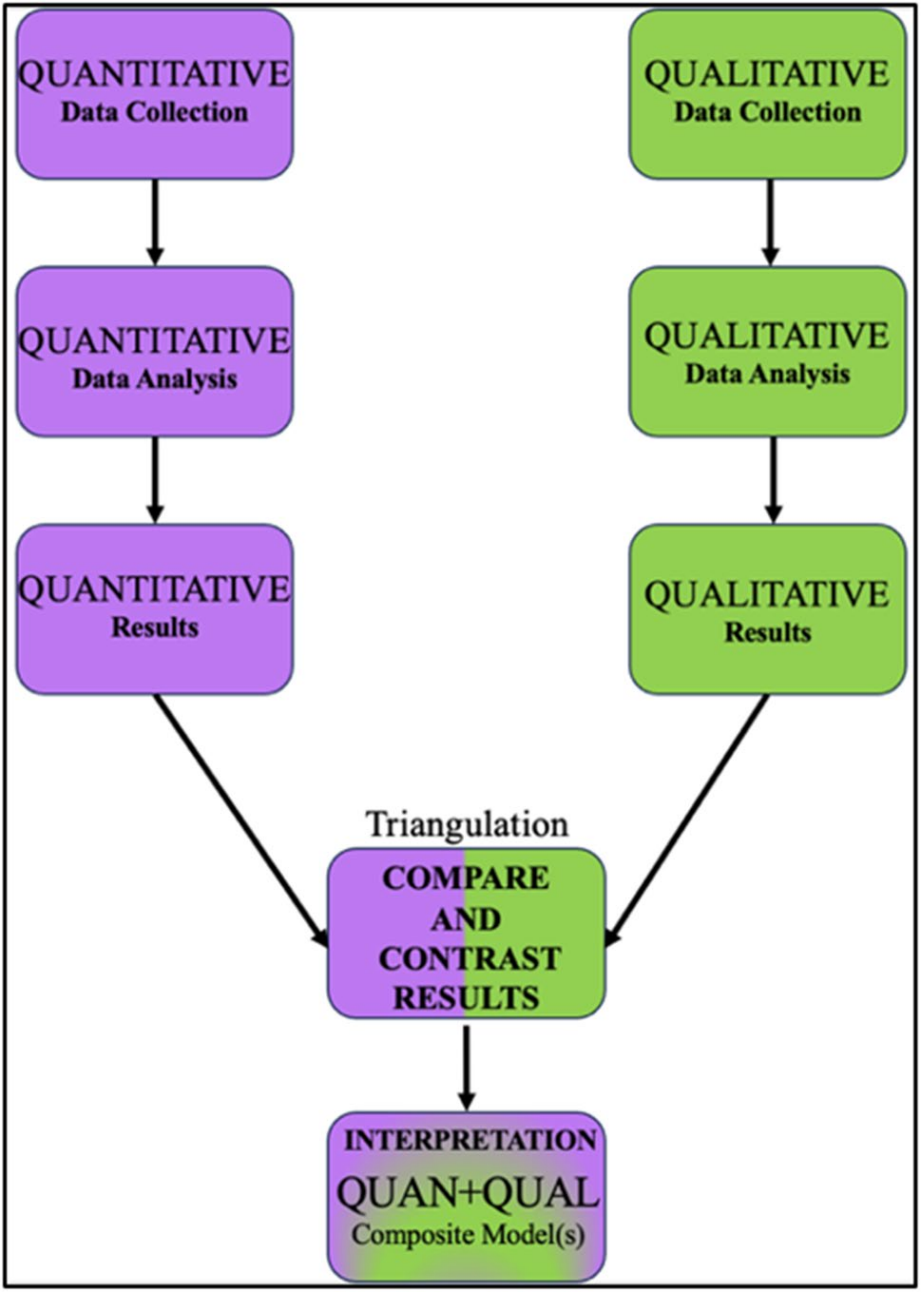
Convergent parallel mixed methodology (Creswell and Clark (2017) and Creswell (2009))

This study was conducted under a convergent parallel mixed-methods design (Fig. 1). The quantitative phase utilized a pretest/posttest design using the Professionalism Assessment Scale (PAS) [7]. The continuous day-to-day activities and experiences in the gross anatomy laboratory (e.g., showing up to lab on time, behaving in accord with ethical principles, care, and treatment of cadaveric donors) over the course of the semester were considered the interventions for the professionalism competency which occurred between the pre- and posttests. The qualitative phase used student competency development portfolios (CDPs) developed for the competency-based curriculum in the Clinical Anatomy, Development, and Examination (CADE) course at ULSOM as data materials. These portfolio entries were thematically analyzed using coding and grounded theory principles [8–10] to describe how students perceive the development of their Professionalism competency over the CADE course. The results from each phase of the study were then triangulated to give a holistic description of first-year medical student professionalism competency development over time in the gross anatomy lab context.

### Quantitative Materials and Methodology

First-year medical students at ULSOM completed quantitative self- and peer-assessments of professionalism skills using the previously validated 25-item Behavioral Professionalism Assessment, the Professionalism Assessment Scale (PAS) adapted from Hammer et al. [7] at the beginning and at the end of the gross anatomy course (*n* = 83). The PAS was developed as an instrument to assess behavioral professionalism of pharmacy students [7]. The PAS was found to be psychometrically sound based on measures of internal consistency (Cronbach’s *α* = 0.973), factor analysis, and interscale correlations when used with pharmacy preceptors and their students participating in experiential rotations [7]. The PAS assesses specific behavioral attributes of Professionalism under four main domains: Interpersonal Relations/Social Skills, Responsibility, Professional Communication, and Appearance. The researchers encouraged testing the instrument in other professional education environments to strengthen external validity, and that none of the scale items, if deleted from the instrument, would drastically reduce the overall Cronbach’s *α* of 0.973 [2]. We adapted the 25-item PAS to assess first-year medical students’ professionalism skills in the gross anatomy lab context. The PAS for the gross anatomy lab context consists of 24 items scored on a Likert scale anchored by 1 (unsatisfactory) and 5 (excellent). Self-assessment (PAS-SA-1 and PAS-SA-2) and peer-assessment (PAS-PA-1 and PAS-PA-2) versions were created.

The quantitative phase of this study included several statistical analyses including an internal reliability assessment of the PAS-self-assessments (PAS-SAs) and PAS-peer-assessments (PAS-PAs), dependent paired samples *t*-tests of the self- and peer-assessment data, and measures of effect size. Data were analyzed within SPSS version 29.0, with a significance level of* p* < 0.05. Before conducting any statistical analyses, the data were examined to ensure that the assumptions of the statistical tests were met.

### Qualitative Materials and Methodology

The students completed three competency development portfolio (CDP) assignment entries throughout the CADE course (*n* = 83). The first entry at the beginning of the semester asked students to reflect on their strengths and weaknesses within the Professionalism competency and to develop SMART goals [11] for the competency. The second entry at mid-semester asked students to reflect on and track their progress in the Professionalism competency thus far and to revise or set new goals in the competency for the remainder of the semester. The third entry at the end of the semester asked students to reflect on their progression in the Professionalism competency throughout the semester and to make a plan for how they will continuously maintain and improve their skills in the competency as it relates to the medical field.

Thematic analysis and grounded theory principles were used to qualitatively analyze the students’ responses in their CDPs in the qualitative data analysis software MaxQDA version 22.5.0. Students’ CDP part 1 entries were initially read, then coded, and then axially coded to find the main themes, and categories within those themes, present in first-year medical students’ strengths and weaknesses and goals set within the Professionalism competency at the beginning of the semester. Students’ CDP part 2 entries were then read, coded, and then axially coded to find the main categories and themes present in students’ progress in and reflections on their Professionalism competency goals set at the beginning of the semester. Students’ CDP part 3 entries were then read, coded, and axially coded to find the main categories and themes present in the students’ summative reflection on their overall development within the Professionalism competency over the CADE course.

CDP entries were coded until it was determined that inductive thematic saturation [12, 13] was reached for the main categories and themes that erupted from the data during constant comparison of the dataset. Once this coding process was complete, selective coding across all created categories was used to determine the most common themes and categories students discussed within their professionalism competency development over the CADE course. These themes and their categories were then used to generate theories and propositions that describe the interrelationship of the themes and categories to describe student perceptions on professionalism skill development in the gross anatomy lab context over time.

### Timeline and Gross Anatomy Lab Setting

**Fig. 2:**
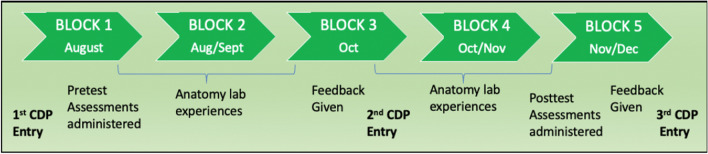
Organization of CADE course and timeline of administration of assessments and CDPs

At the start of the CADE course, first-year medical students were assigned to a cadaveric donor at a table within a zone in the gross anatomy lab. Six to seven students were assigned to each donor, and those six to seven students were further subdivided into teams of three to four students. The students were in these teams all semester at the same table and with the same cadaveric donor, and they alternated dissection days throughout the semester (30 lab sessions total). During Block 1 of the CADE course in August of 2022, after dissecting together once or twice, these teams of three to four students were asked to self-assess (PAS-SA-1) and peer-assess (PAS-PA-1) their two to three dissection teammates (Pretest Assessments), and to complete CDP entry 1. At mid-semester in Block 3, students were provided with faculty and peer feedback for the Professionalism competency based on the pre-assessments from Block 1. Assessment plus feedback is one of the central tenets of CBME, and feedback is considered necessary for students to further develop skills within a competency domain [14, 15]. After receiving this feedback, the students completed their second CDP entry. During Block 5, the dissection teams were asked to self-assess (PAS-SA-2) and peer-assess (PAS-PA-2) their two to three dissection teammates (Posttest Assessments). After the post-assessments were administered, students received a final round of faculty and peer feedback based on the post-assessments and then completed their final CDP entry. Refer to Fig. 2 for a visual description of this timeline and methodology.

## Results

### Reliability Analyses

A Cronbach *α* reliability analysis with an intraclass correlation coefficient (ICC) confidence interval estimate was conducted to measure the internal consistency/reliability of the scale items on each version of the PAS. Cronbach’s *α* and ICC estimates and their 95% confidence intervals were calculated using SPSS version 29.0 based on a two-way mixed-effects model [16, 17]. For the PAS-SA-1, the obtained ICC/*α* value is 0.875 (indicating good reliability); its 95% confidence interval ranges between 0.833 and 0.911 (indicating good to excellent reliability). For the PAS-SA-2, the obtained ICC/*α* value is 0.918 (indicating excellent reliability); its 95% confidence interval ranges between 0.891 and 0.942. For the PAS-PA-1, the obtained ICC/*α* value is 0.901; its 95% confidence interval ranges between 0.878 to 0.922. For the PAS-PA-2, the obtained ICC/*α* value is 0.924; its 95% confidence interval ranges between 0.906 and 0.939. Table 1 illustrates these results.

**Table 1:** Cronbach’s alpha and ICC results for the PAS

	Cronbach’s alpha	Intraclass correlation	95% confidence interval		*N* of items
			Lower bound	Upper bound	
PAS-SA-1	.875	.875	.833	.911	24
PAS-SA-2	.918	.918	.891	.942	24
PAS-PA-1	.901	.901	.878	.922	24
PAS-PA-2	.924	.924	.906	.939	24

### Scale Items and Statistics

Tables 2 and 3 illustrate the items on the PAS-SAs and PAS-PAs and their respective descriptive statistics.

**Table 2:** Item statistics for PAS-SAs

	PAS-SA-1		PAS-SA-2		*N*
	Mean	Std. deviation	Mean	Std. deviation	
Student is reliable and dependable	4.80	.435	4.88	.363	83
Student produces quality work	4.48	.687	4.77	.423	83
Student is empathetic	4.92	.320	4.90	.297	83
Student behaves in an ethical manner	4.93	.261	4.92	.280	83
Student communicates articulately	4.54	.570	4.71	.456	83
Student is punctual	4.84	.427	4.82	.417	83
Student uses time efficiently	4.71	.530	4.83	.408	83
Student is self-directed in undertaking tasks	4.71	.482	4.81	.397	83
Student maintains confidentiality	4.94	.239	4.92	.280	83
Student is respectful	4.95	.266	4.95	.215	83
Student communicates using appropriate body language	4.87	.341	4.90	.297	83
Student demonstrates accountability	4.92	.280	4.92	.280	83
Student prioritizes responsibilities effectively	4.76	.458	4.86	.354	83
Student accepts and applies constructive criticism	4.81	.426	4.82	.417	83
Student puts others’ needs above their own	4.71	.482	4.76	.430	83
Student is nonjudgmental	4.92	.280	4.84	.366	83
Student communicates assertively	4.55	.685	4.72	.477	83
Student is an active learner	4.81	.397	4.90	.297	83
Student is cooperative	4.95	.215	4.95	.215	83
Student is diplomatic	4.92	.280	4.86	.387	83
Student “follows through” with responsibilities	4.87	.341	4.90	.297	83
Student wears appropriate attire	4.94	.239	4.96	.188	83
Student demonstrates confidence	4.61	.559	4.69	.583	83
Student demonstrates a desire to exceed expectations	4.61	.601	4.72	.450	83

**Table 3:** Item statistics for PAS-Pas

	PAS-SA-1		PAS-SA-2		*N*
	Mean	Std. deviation	Mean	Std. deviation	
Student is reliable and dependable	4.88	.359	4.94	.283	169
Student produces quality work	4.80	.483	4.90	.339	169
Student is empathetic	4.91	.285	4.91	.342	169
Student behaves in an ethical manner	4.93	.300	4.97	.170	169
Student communicates articulately	4.83	.450	4.93	.280	169
Student is punctual	4.87	.483	4.83	.496	169
Student uses time efficiently	4.91	.332	4.93	.280	169
Student is self-directed in undertaking tasks	4.83	.484	4.89	.379	169
Student maintains confidentiality	4.99	.108	4.99	.077	169
Student is respectful	4.94	.261	4.96	.186	169
Student communicates using appropriate body language	4.95	.213	4.96	.186	169
Student demonstrates accountability	4.93	.270	4.96	.265	169
Student prioritizes responsibilities effectively	4.91	.349	4.93	.280	169
Student accepts and applies constructive criticism	4.89	.363	4.92	.289	169
Student puts others’ needs above their own	4.80	.454	4.87	.402	169
Student is nonjudgmental	4.94	.237	4.94	.261	169
Student communicates assertively	4.83	.476	4.94	.261	169
Student is an active learner	4.90	.321	4.95	.239	169
Student is cooperative	4.96	.186	4.96	.200	169
Student is diplomatic	4.92	.309	4.96	.228	169
Student “follows through” with responsibilities	4.92	.297	4.96	.215	169
Student wears appropriate attire	4.99	.108	4.99	.077	169
Student demonstrates confidence	4.83	.500	4.86	.440	169
Student demonstrates a desire to exceed expectations	4.77	.476	4.92	.335	169

### Quantitative: PAS-SA Dependent t-Test

The PAS-self-assessment was administered at pretest (PAS-SA-1) and sought to measure self-assessed levels of professionalism skills at the beginning of the semester. The PAS-self-assessment was utilized again at posttest (PAS-SA-2) and sought then to measure self-assessed levels of professionalism skills at the end of the semester. A dependent paired samples *t-*test on the pretest/posttest data for the PAS-self-assessment was utilized to determine whether change was self-perceived by first-year medical students. There was no statistical difference in PAS-self-assessment scores from Time 1 (*M* = 4.81, *SD* = 0.209) to Time 2 (*M* = 4.85, *SD* = 0.217), *t* (82) =  − 1.623, *p* = 0.108 (two-tailed). The mean difference in PAS-self-assessment scores was 0.034 with a 95% confidence interval ranging from − 0.075 to − 0.008. Cohen’s *d*, a measure of effect size, showed a small effect (0.1895). Tables 4, 5, and 6 illustrate these results.

**Table 4:** Paired samples statistics for PAS-SAs

	Mean	*N*	Std. deviation	Std. error mean
PAS-SA-1	4.81	83	.209	.023
PAS-SA-2	4.85	83	.217	.024

**Table 5:** Paired samples test for PAS-SAs

	Paired differences					*t*	df	Significance two-sided *p*
	Mean	Std. deviation	Std. error mean	95% confidence interval of the difference				
				Lower	Upper			
PAS-SA-1 to PAS-SA-2	− .034	.190	.021	− .075	− .008	− 1.623	82	.108

**Table 6:** Paired samples effect sizes for PAS-SAs

		Standardizer	Point estimate	95% confidence interval	
				Lower	Upper
PAS-SA-1 to PAS-SA-2	Cohen’s *d*	.1895	− .178	− .394	.039

### Quantitative: PAS-PA Dependent t-Test

The PAS-peer-assessment was utilized at pretest (PAS-PA-1) and sought to measure levels of professionalism skills of peers at the beginning of the semester. The PAS-peer-assessment was utilized again at posttest (PAS-PA-2) and sought then to measure levels of professionalism skills of peers at the end of the semester. A dependent paired samples *t-*test on the pretest/posttest data for the PAS-peer-assessment was utilized to determine whether change is peer-perceived by first-year medical students. There was a statistically significant increase in PAS-peer-assessment scores from Time 1 (*M* = 4.89, *SD* = 0.165) to Time 2 (*M* = 4.93, *SD* = 0.127), *t* (82) =  − 2.890, *p* = 0.005 (two-tailed). The mean difference in PAS-peer-assessment scores was 0.040 with a 95% confidence interval ranging from − 0.068 to − 0.012. Cohen’s *d*, a measure of effect size, showed a negligible effect (0.127). Tables 7, 8, and 9 illustrate these results.

**Table 7:** Paired samples statistics for PAS-Pas

	Mean	*N*	Std. deviation	Std. error mean
PAS-PA-1	4.89	83	.165	.018
PAS-PA-2	4.93	83	.127	.014

**Table 8:** Paired samples test for PAS-Pas

	Paired differences					*t*	df	Significance two-sided *p*
	Mean	Std. deviation	Std. error mean	95% confidence interval of the difference				
				Lower	Upper			
PAS-PA-1 to PAS-PA-2	− .040	.127	.014	− .068	− .012	− 2.890	82	.005

**Table 9:** Paired samples effect sizes for PAS-Pas

		Standardizer	Point estimate	95% confidence interval	
				Lower	Upper
PAS-PA-1 to PAS-PA-2	Cohen’s *d*	.127	− .317	− .537	− .096

### Qualitative Results

Thematic analysis with principles of grounded theory was utilized to examine student reflections on their baseline status and goal setting, progress, and development within the Professionalism competency during the CADE course. The goal of this analysis was to explore and contextualize the personal experiences students had while developing their professionalism competency skills during the CADE course.

The qualitative results revealed three main themes that coursed throughout all three parts of the CDP: Interpersonal Relations/Social skills, Responsibility skills, and Gross Anatomy lab skills. These themes involved skills within the Professionalism competency that students discussed strengths and weaknesses within and set goals for improving throughout the semester.

## Interpersonal Relations/Social Skills Development

For students who discussed aspects of Interpersonal Relations/Social skills, at the beginning of the semester, there was a focus on their strengths and weaknesses in respecting others and peer-to-peer professionalism. Students noted strengths in respecting others’ time and treating others with respect, while some noted weaknesses in respecting others by interjecting when others are speaking or not giving others proper attention while they are speaking. An aspect of respect is honesty and accountability, and many students discussed weaknesses in acknowledging when they make mistakes and speaking up if a mistake is made. Students set goals around admitting to mistakes if they occur, keeping eye contact with others to give them full attention when they are speaking, and being honest with and expressing feelings to their teammates when needed. Students also discussed weaknesses with peer-to-peer professionalism, like lacking professionalism around peers because “working with peers and hanging out with friends is a fine line” and avoiding taking action if a peer is acting unprofessionally, with students specifically noting being weaker in addressing others’ inappropriate comments in the gross anatomy lab. These students set goals to uphold their peers to a higher standard of professionalism and to take action on unprofessional peer behavior if it occurs. Table 10 illustrates representative quotations regarding Interpersonal Relations/Social Skills Development from CDP part 1.

**Table 10:** Representative quotations from CDP 1 regarding interpersonal relations/social skills development

“A strength I have in professionalism is **showing respect** to my teammates by listening before speaking, always making eye contact, never raising my voice with anyone.” ID64 “One of my weaknesses in this competency is that I am often **very worried about messing up**. This leads to **not speaking up** if I feel that **I've made a mistake** and **just trying to fix it on my own**.” ID27 “Weakness- it's **easy to lose sight of the task** at hand because **working with peers and hanging out with friends** is **a fine line**” ID35	

At mid-semester, there was a shift within this theme from a self-directed focus to a team-directed focus on interpersonal relations/social skills. In the first CDP, students discussed being honest with and accountable to *themselves* in advancing professionalism, as well as working toward *personally* addressing unprofessionalism of others. By contrast, in CDP entry 2, students reflected more on their own individual professionalism goals of their team and toward their team. The tone of students’ reflections clearly shifted from a “me” to a “we” perspective, with students noting how they believed their teams treat each other with respect, honesty, and accountability, and how their teammates work well together and maintain professional, positive attitudes. Students created goals involving how to treat their teammates professionally for the remainder of the semester. These included goals like giving teammates their full attention, confronting teammates privately if there is a conflict, asking teammates for feedback on how to improve, and having teammates hold them accountable for their goals. Table 11 illustrates representative quotations regarding Interpersonal Relations/Social Skills Development from CDP part 2.

**Table 11:** Representative quotations from CDP 2 regarding interpersonal relations/social skills development

“**We've done a fantastic job** of making sure that **each member receives appropriate** and **equal praise** and **feedback** for dissections well done.” ID55 “I feel as though **we were all at around the same level** of **maintaining professionalism**, especially when necessary.” ID28 “**Give teammates my full attention when they are talking to me**…I will ask my teammates to hold me accountable for this goal. If they see that I haven't stopped to give my full attention, then they can bring it to my attention that I am not actively engaged in conversation. I will **identify** my **success by decreasing the amount of time my team members have to call out my behavior**.” ID9	

**Fig. 3:**
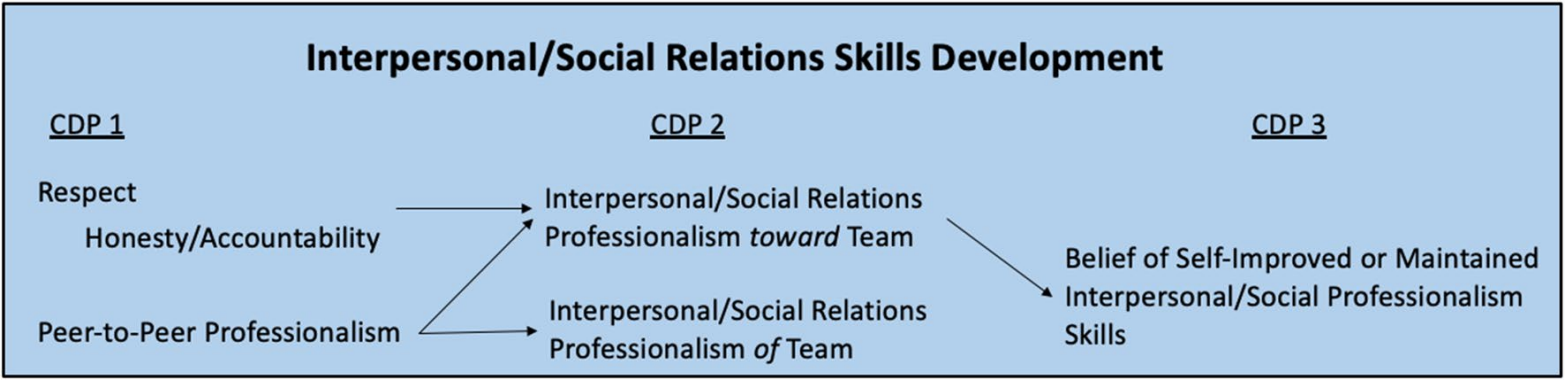
Summary of first-year medical students’ process of Interpersonal/Social Relations Skills Development over the course of three CDP entries

By the end of the semester, several students mentioned how they believed they maintained a consistent level of professionalism from the beginning of the course to the end of the course. Some students discussed beliefs of improving aspects of interpersonal relations/social skills by making conscious efforts to give others their full attention when speaking, learning about professionalism in group settings with peers, getting used to working with the same team, and treating team members with respect. Table 12 illustrates representative quotations regarding Interpersonal Relations/Social Skills Development from CDP part 3. Figure 3 provides a summary of the first-year medical students’ process of Interpersonal Relations/Social Skills development over the course of the semester.

**Table 12:** Representative quotations from CDP 3 regarding interpersonal relations/social skills development

“I believe my **professionalism** at the beginning of the semester was **adequate** as I show up on time, **treat my colleagues with respect, and am honest when making a mistake.** I did not have any issues with my professionalism competency at the beginning of the semester, and throughout the semester **I maintained this competency**.” ID20 “**I have improved my professionalism skills** since the beginning of this semester by **learning much more about professionalism in group settings** especially when it comes to **working with peers**.” ID53

## Responsibility Skills Development

For students who discussed aspects of the Responsibility skills, at the beginning of the semester, there was a focus on time management and organizational skills, including punctuality to lab sessions. Several students indicated strengths in punctuality, showing up early and being ready to go, while many students indicated weaknesses in being punctual and arriving right on time to scheduled events. These students set goals for how to improve their difficulties with punctuality, like leaving their houses earlier, showing up several minutes early to scheduled lab sessions, and asking peers to hold them accountable if they are ever late. Students also mentioned some difficulties with organizational skills like staying on task, forgetting details, and being overwhelmed due to not being very organized. These students had goals involving keeping a calendar or planner and looking at it daily and keeping track of tasks in lists and within calendars. Table 13 illustrates representative quotations regarding Responsibility Skills Development from CDP part 1.

**Table 13:** Representative quotations from CDP 1 regarding responsibility skills development

“My biggest weakness regarding professionalism is **punctuality**. I have always been a person that **runs 5–10 min late**, and in **medical school that is no longer acceptable**. I want to ensure I am **managing my time wisely** and show up to every lab on time ready to work.” ID16 “Weaknesses: **I find myself feeling overwhelmed** because **I am not as organized** as I would like” ID14 “My goal would be **to get a calendar** that just tracks assignments and due dates, success would be no small assignment gets forgotten.” ID75	

By mid-semester, several of these students had assumed a more self-perceived control of the responsibility domain, citing achieving mastery or acting intentionally toward goals in this area, specifically in regard to successful time management resulting in punctuality to lab and planner/calendar organization resulting in being less overwhelmed and keeping track of all tasks. Another subset of students, however, at mid-semester realized they were struggling with the responsibility domain. These students created new goals surrounding time management for the remainder of the semester. These goals consisted of actions like going to bed earlier, waking up earlier, prepping lunch the night before rather than morning of, showing up several minutes early, and being ready to go by the start of the lab session. Students recognized needing to show up earlier rather than right on time so that their teammates did not need to do all of the setup work prior to lab starting, like grabbing an iPad with the dissection guide for the day. Table 14 illustrates representative quotations regarding Responsibility Skills Development from CDP part 2.

**Table 14:** Representative quotations from CDP 2 regarding responsibility skills development

“I believe **I have mastered this goal** due to **not being overwhelmed this semester**. **I feel very organized** from previous years. I have been **actively checking my planner** twice each week.” ID18 “I have **intentionally worked on my professionalism competency** as I have still **never been late to lab** and actively try to leave my home at least an hour before lab begins” ID3 “While I am slightly earlier for labs than I was before CDP Part One**, I would like to push myself** to be even **earlier**. I am now about 5 or so minutes early. **I would like to push myself to be at least 15 min early to lab** to ensure that my group has adequate time to review and prepare prior to dissection.” ID55	

**Fig. 4:**
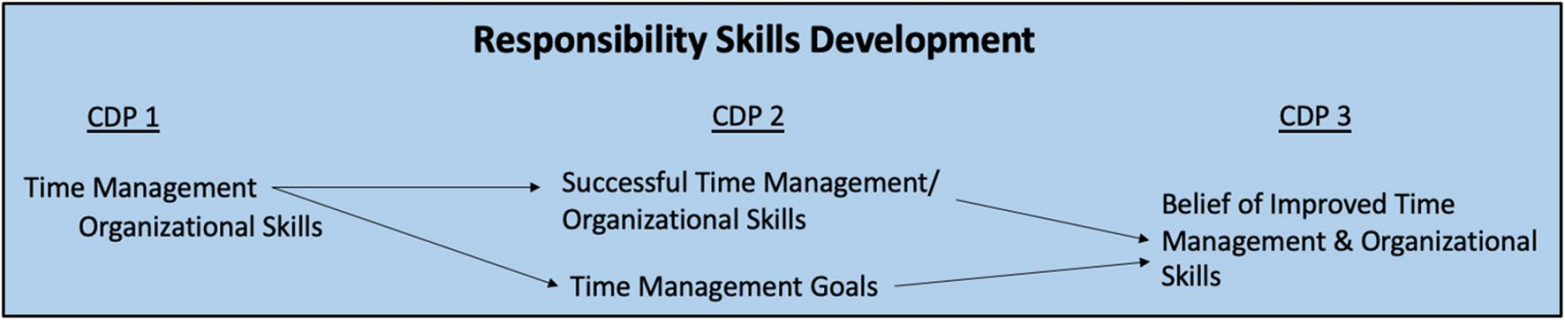
Summary of first-year medical students’ Responsibility Skills Development over the course of the three CDP entries

By the end of the semester, students who reflected on their Responsibility skills claimed they believed they improved their skills over the course of the semester by getting better at arriving earlier rather than right on time, saying “no” to potential distractions from studies, and keeping track of small details and responsibilities in a more organized way. Some students noted how they still had room for improvement within this domain, whether that was to get better at showing up earlier to events or recognizing that juggling multiple deadlines and responsibilities will continue and thus developing a mechanism for managing time most effectively will be crucial moving forward. Table 15 illustrates representative quotations regarding Responsibility Skills Development from CDP part 3. Figure 4 provides a summary of the first-year medical students’ process of Responsibility Skills development over the course of the semester.

**Table 15:** Representative quotations from CDP 3 regarding responsibility skills development

“I originally wrote about keeping up with the small details **and ensuring that I keep track of all responsibilities**. **I accomplished this goal** thanks to a new calendar and notebook for writing down details I would be likely to forget. This new system has worked well and allowed me to keep up with many goals I may have forgotten in the past.” ID33 “I **began to manage my time more effectively**, say "no" to anything that could possible distract me from my studies, and allowed myself to have some more personal/individual time.” ID18 “I believe that **I showed professionalism throughout the semester** and was always **intentional about showing up on time**.” ID71

## Gross Anatomy Lab Skills Development

For students who discussed Gross Anatomy lab-specific professionalism skills, at the beginning of the semester, the students focused on personal respect for the cadaveric donors and preparation for lab sessions. Many students recognized how they consistently treat the donors with respect and act professionally around and toward the donors, while some students noted how they can sometimes be careless in the lab setting or make inappropriate remarks or use inappropriate language when dissecting the donors. These students acknowledged the need to understand that the lab is a professional environment and to use language that fits that environment, while other students noted that if they hear their peers make disrespectful comments toward or around the donors that they will address their peers respectfully but hold them accountable to being appropriate around the donors at all times. Students also discussed preparation for lab sessions, with many noting that they are not always fully prepared for lab in that they did not fully review the pre-lab guides or lectures prior to their dissection days. Several students set goals surrounding being better prepared for lab by doing the pre-lab a few days ahead of time, reviewing lecture material for lab days in advance, and taking notes on the dissection guides so that their dissections progress more efficiently and they feel more confident with the material when they step foot into the lab. Table 16 illustrates representative quotations regarding Gross Anatomy Lab Skills Development in CDP part 1.

**Table 16:** Representative quotations from CDP 1 regarding gross anatomy lab skills development

“I **will act professionally by more thoroughly going over lab guide prior to lab** and drafting up a plan for efficient dissection with group members.” ID37 “Weakness: I will sometimes **use "colorful" language when there is a part of dissection that is jarring**. For instance, when dissecting the hand, there was what looked like blood that came out as I was skinning and that was particularly challenging for me.” ID31 “One **weakness** of mine when it comes to professionalism is **always being completely prepared**. There have been some labs where I did not have enough time to really learn the lab ahead of time and would need to consult the manual a lot during lab.” ID53	

At mid-semester, some students reflected on how they believed they were doing a better job at preparing for lab, having a stronger understanding of the anatomic relationships prior to dissecting them which increased confidence and comfortability in the lab setting. Other students reflected on how they were still struggling to consistently prepare for lab ahead of time. As students set or revised their goals for the remainder of the semester, they planned to read the dissection guides thoroughly the day before each dissection, become more comfortable with lecture content, and show up to lab with pre-annotated checklists to indicate they had prepared prior to lab. Students noted that being more prepared will contribute to helping their dissection team work through the content together. Several students also reflected on how they had treated their cadaveric donors thus far in the semester, with the majority stating they were taking proper and appropriate care of their donor and cleaning their lab areas. It was also noted that thorough preservation of the donors was sometimes lacking, and “as keepers of our donor’s temporary resting place and remains, I want to do a better job caring for them.” Therefore, some students set goals for honoring their donors throughout the remainder of the semester. These goals included being a better steward to the donors by ensuring proper maintenance using appropriate wetting techniques, holding others accountable for respecting the donors, and being more conscientious in the lab environment by avoiding making jokes. Table 17 illustrates representative quotations regarding Gross Anatomy Lab Skills Development in CDP part 1.

**Table 17:** Representative quotations from CDP 2 regarding gross anatomy lab skills development

“One way that **I can be more professional is to come as prepared as possible to lab** before the dissection occurs. I have a tendency to skim through the lab guides before bed the night before a dissection. I will make sure that I **read the lab guides thoroughly** the day **before each dissection** during the last block” ID71 “A new goal that I would like to set is **holding other peers accountable for respecting the donors**. I will achieve this goal by politely and privately **addressing a classmate who makes an inappropriate comment** and **explaining to them why it is disrespectful**.” ID13 “**I can improve my skills in the professionalism competency** by **keeping in mind the environment** and context in that **the body was graciously donated to help us as students**. **Sometimes it's easy to forget that** and jokes can be made. **I will be more conscientious in the lab environment**.” ID3	

**Fig. 5:**
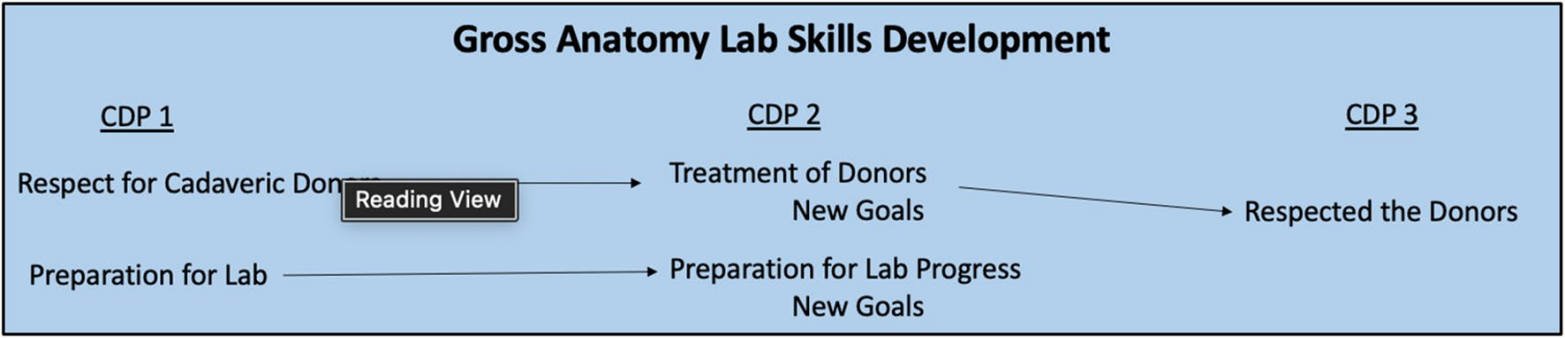
Summary of first-year medical students’ Gross Anatomy Lab Skills Development over the course of three CDP entries

By the end of the semester, students who reflected on their gross anatomy lab-specific professionalism skills noted how they took their time in lab seriously and acknowledged the magnitude of the donors’ gift, being proud of handling their donor with respect even with being timid about working with the donor at the start of the semester, and improving language use in the lab and other professional settings. It was noted that “working with the donor taught me, in addition to anatomy, how to properly care for my future patients” (Table 18). Table 18 illustrates representative quotations regarding Gross Anatomy Lab Skills Development from CDP part 3. Figure 5 provides a summary of the first-year medical students’ process of Gross Anatomy Lab Skills development over the course of the semester.

**Table 18:** Representative quotations from CDP 3 regarding gross anatomy lab skills development

“I **took our time in the lab seriously**, and the **magnitude of our donors' gift of themselves wasn't lost on me**.” ID60 “Going into medical school, I was most tepid about the willed body lab**. I did not know how to react to seeing a body** and **working with that body**. After our semester of lab**, I am most proud of how I handled my donor with respect**. I think **working with the donor taught me, in addition to anatomy, how to properly care for my future patients**.” ID23 “I **started off the year without much of a filter in lab**. When I saw something shocking or alarming I would say the first words that came to mind, which weren't always the most professional. **I have grown so much in this skill throughout the year**…**I know I have developed this skill because I now pause when I experience an unexpected situation and collect myself before speaking**.” ID31

### Triangulation

These results from the quantitative and qualitative methods were triangulated to determine if convergence occurred or if discrepancies were observed between datasets. Methodological triangulation was useful and necessary to address multiple aspects of the same phenomenon — professionalism competency development over time in the gross anatomy lab context. This triangulation aimed to address the following questions:

- Do first-year medical students exhibit statistical growth over time? If so, how? If not, why?
- What skills do students already possess in this competency, what skills did they believe they gained or improved upon?
- How do students feel about their progression in the competency?

The quantitative assessments provided static snapshots of students’ behavioral professionalism skills and the opportunity to determine if growth was self- and/or peer-perceived, while the open-ended qualitative portfolio entries furnished the opportunity for longitudinal exploration of student perspectives on their progression through behavioral attributes of professionalism in the gross anatomy lab context.

**Fig. 6:**
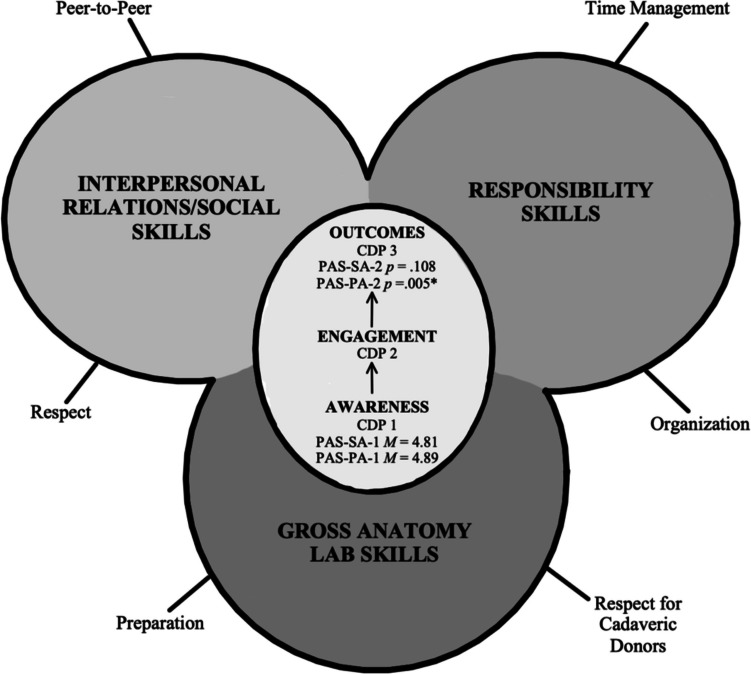
Composite model of Professionalism Competency Development in the ULSOM Gross Anatomy Laboratory context

The quantitative aspect of this study demonstrated that first-year medical students at the ULSOM did not exhibit statistically significant growth from PAS-SA-1 to PAS-SA-2 (*p* = 0.108). There are several reasons why this finding may have occurred. Within the CDPs, when students reflected upon their strengths and weaknesses at the start of the semester, many students indicated that professionalism was already a strength coming into medical school and resulting in students ranking themselves very highly [18] on the PAS early in the semester (*M* = 4.81). As they progressed through the semester, students noted how they felt like they had maintained a high level of professionalism ever since the beginning of the semester. This likely contributed to students ranking themselves at a similarly high level on the PAS at the end of the semester as they did at the beginning of the semester (*M* = 4.85), resulting in no statistically significant growth or decline and rather a high maintenance level of professionalism skills. While overall statistically significant growth was not found between PAS-SA-1 and PAS-SA-2 (Fig. 6), a subset of students discussed how they interpreted they had improved Responsibility skills such as time management and organization over the course of the semester, and when directly comparing the Responsibility item statistics from the PAS-SAs, students did demonstrate increased skill in using time efficiently (*M* = 4.71 to *M* = 4.83) and in being self-directed in undertaking tasks (*M* = 4.71 to *M* = 4.81) (Table 2).

The quantitative aspect of this study also demonstrated that first-year medical students at ULSOM perceived their peers to have exhibited statistically significant growth on the PAS-PA (*p* = 0.005) (Fig. 6). When looking at the literature, this result is consistent with previous medical education Professionalism self- and peer-assessment studies that found that students tend to underrate themselves compared to ratings received from peers [6, 19]. Students self-assessed themselves lower than their peers assessed them at the beginning (Self *M* = 4.81/Peer *M* = 4.89) and at the end of the semester (Self *M* = 4.85/Peer *M* = 4.93). A possible reason for this difference in our study is showcased in our qualitative results. In CDP part 1, students mainly focused on intrinsic professionalism skills, weaknesses, and goals, and by CDP part 2, some students had shifted to discussing the successful professional attributes of their team as a whole. This unprompted recognition and reflection on the professionalism of peers could indicate why we saw statistically significant growth from PAS-PA-1 to PAS-PA-2. Figure 6 provides the composite model of first-year medical students’ awareness, engagement, and outcomes of their Professionalism Competency Development in the ULSOM Gross Anatomy Laboratory context in the fall of 2022.

## Discussion

This study used quantitative self- and peer-assessments along with qualitative self-authored longitudinal portfolio reflections to investigate professionalism competency development of first-year medical students in the gross anatomy lab context over time. The quantitative results indicated that first-year medical students at ULSOM self-assessed professionalism at a very high level (> 4.8) [18] and they did not exhibit self-assessed growth from the beginning of the semester to the end of the semester. The quantitative results also demonstrated that students assessed their peers at a very high baseline level (> 4.85) [18], and that students perceived that their peers had improved professionalism skills from the beginning of the semester to the end of the semester.

The qualitative results revealed three main themes that coursed throughout all three entries of the CDP over time: Interpersonal Relations/Social skills, Responsibility skills, and Gross Anatomy lab specific professionalism skills. These themes involved many skills within the professionalism competency that students had personal strengths and weaknesses within and set goals for improving throughout the semester. They reflected on progression through those goals and stated how they believed they either maintained, were still working on, or improved upon their skills within these themes since the beginning of the semester.

While the qualitative analysis gave more meaningful inferences in comparison to the quantitative data, overall, the qualitative results combined with the quantitative results gave us a more holistic picture of first-year medical student Professionalism competency development over time in the gross anatomy lab context. We have found that students in this context self-assess and peer-assess at a consistently high level [18], and that students perceive their peers to have increased professionalism skills over the course of the semester. We have also found that students have an overall belief of either maintaining their already high skills within professionalism over the course of a semester, or of improving certain aspects of the competency such as organizational and time management skills. Since peer-assessment has been found to be valid and reliable [6, 20, 21], combining it with self-assessment and self-reflection provides a holistic form of multisource feedback (MSF) to provide students with an understanding of what is expected of them and what to expect of themselves professionally in the first year of medical school in the gross anatomy lab context. 

This study also indicates what qualities first-year medical students consider the most relevant when asked open-ended portfolio questions regarding the professionalism competency in the gross anatomy lab context. Respect, maintaining and balancing professionalism with medical student peers, proper time management, organization, and punctuality, honoring and respecting cadaveric donors, and preparing appropriately for lab were the aspects of professionalism that students acknowledged most frequently.

Students identifying the importance of honoring and respecting cadaveric donors and understanding that is an aspect of professionalism development indicates that the gross anatomy lab is a feasible venue to incorporate professionalism assessment for first-year medical students. Working with a cadaver for an entire semester inherently poses moral and professional challenges for these students, as the donors embody the students’ “first patient” and they exhibit total dependence on the students just like a patient exhibits dependence on their doctor [4]. As students cannot have a discourse with their donors, they must make critical moral and professional decisions while working with a donor, including what language they use around and when referring to the donor. Recognizing and reflecting upon these moral and professional questions early in their medical education encourage students to learn important professional values that will be directly applicable to their future practice as physicians—like maintaining patient confidentiality and dignity and behaving with respect and integrity [5].

### Limitations

While all attempts were made to minimize potential limitations and potential sources of bias, we acknowledge that this study was limited by many factors. First, the study was conducted at a single undergraduate medical education institution; therefore, these results may not be generalizable to first-year medical students at other medical schools. Further research in this area at other UME institutions is suggested to enhance validation of the results. Other potential limitations in this study are social desirability bias (self- and peer-assessments and CDPs seen by faculty which could have swayed students to respond in a certain manner) and assessment fatigue (students completed 18 total text entries in their CDPs, as well as six self-assessments and 12–18 peer-assessments over the course of the semester).

## Conclusions

The findings of this study should be used to guide the efforts of medical educators in teaching and assessing competencies such as professionalism skills, within the preclinical years of medical education. Dissection-based gross anatomy laboratories with faculty who are willing to implement the education and assessment of nontraditional discipline-independent skills [22] can be the perfect place of opportunity for implementing competency-based assessment to further promote the educational continuity from undergraduate medical education and beyond. This study has demonstrated how the Professionalism competency is inherently present in the gross anatomy lab context, how it can be successfully incorporated into the gross anatomy lab dissection-based curriculum, and how assessing first-year medical students in this competency and in this context is feasible. When students are tasked with setting goals for improving weaknesses in the Professionalism competency, then revising, and reflecting on those goals over the course of the semester, they actively choose to work on those weaknesses and reflect on the skills they developed over the course that they deem are necessary to their future as physicians. These are indications that the gross anatomy lab can be a venue for competency-based assessment in UME that aligns with competency-based assessment in graduate medical education (GME).

## Data Availability

The datasets generated during and/or analyzed during the current study are available from the corresponding author on reasonable request.
